# Does Patient Care Depend on Patients’ Health Behaviors? A Study Investigating the Impact of Empathy Among Future Healthcare Professionals on Their Willingness to Help

**DOI:** 10.5964/ejop.15323

**Published:** 2025-11-28

**Authors:** Julian A. Nasello, Jean-Marc Triffaux

**Affiliations:** 1 Department of Clinical Psychology, University of Liège, Liège, Belgium; 2 Psychiatric Day Hospital “La Clé”, Liège, Belgium; 3 Department of Psychiatry, Medicine, University of Liège, Liège, Belgium; Stanford University, Stanford, USA

**Keywords:** empathy, willingness to help, future healthcare professionals, education, health behaviors

## Abstract

**Objectives:**

This study examines the impact of patients’ healthy and unhealthy behaviors on future healthcare professionals’ willingness to help. Additionally, it also investigates how empathy among future healthcare professionals shapes their willingness to help.

**Methods:**

Three hundred future healthcare professionals completed sociodemographic and empathy questionnaires and evaluated 12 clinical vignettes assessing their willingness to help. The vignettes depicted patients engaging in either healthy or unhealthy behaviors.

**Results:**

Participants reported a greater willingness to help patients displaying healthy behaviors compared to those exhibiting unhealthy behaviors (small effect). A moderate positive association was also observed between empathy and willingness to help. Notably, while affective empathy remained a significant correlate, cognitive empathy showed a stronger association with willingness to help in scenarios involving unhealthy behaviors. Although both gender and grade significantly predicted empathy (with moderate and small effects, respectively), neither variable significantly predicted willingness to help.

**Conclusions:**

The findings demonstrate that patients’ health behaviors influence willingness to help and highlight the role of empathy in shaping these intentions. The study therefore supports integrating targeted empathy-focused training into academic curricula to strengthen empathic and related interpersonal skills among future healthcare professionals.

The willingness to help (WTH) is considered a prerequisite of helping behaviors (Zeng et al., 2022). It is defined as the intention one person has to help someone or others under certain circumstances, which represents an internal precursor to actual helping behavior. WTH is frequently used to assess individuals’ propensity to assist a particular target in ethically sensitive experimental contexts. Accordingly, WTH is a preliminary factor positively correlated with helping behaviors (Yan et al., 2012). In a clinical context, helping behaviors — understood as a crucial form of prosocial behavior involving actions intended to assist another person with a problem or to alleviate their distress (Stukas & Clary, 2012) — are central to professional care, as patients seek healthcare providers to receive appropriate care. Such behaviors are inherently other-oriented and beneficial to the recipient (Mason, 2021).

WTH aligns conceptually with the intentions described in the *Theory of Planned Behavior and Reasoned Action* (Ajzen, 1985; Ajzen, 1991; Fishbein & Ajzen, 1975), which posit that behaviors are driven by intentions, attitudes (i.e., beliefs about a behavior), and subjective norms (i.e., beliefs about others’ attitudes toward a behavior). Intentions are seen as immediate precursors of behavior and are shaped by two main factors: an individual’s beliefs about the behavior itself and their beliefs about others’ expectations (Madden et al., 1992). The Theory of Planned Behavior extends this framework by incorporating perceived behavioral control, reflecting individuals’ sense of control over the performance of a behavior. This addition not only impacts intentions but also influences the implementation of those intentions. Therefore, as shown by Wurthmann (2014), attitudes toward behavior predict intentions to engage in that behavior.

In the present study, we targeted WTH (similar to intentions from *Theory of Planned Behavior and Reasoned Action*) not behaviors (actual helping behaviors) because they were difficult to apprehend for ethical reasons. Our aim was to determine how external factors and individual characteristics shape these intentions (i.e., WTH).

As with helping behavior and altruism, WTH varies substantially across contexts (Berkowitz, 1987). Currently, there is limited evidence that healthcare professionals influence patients’ health behaviors (e.g., Heszen-Klemens & Lapińska, 1984), or that patients’ behaviors affect their treatment outcomes (e.g., Jeon et al., 2020; Pinto & Trunzo, 2005). However, to our best knowledge, there is no data showing how patients’ health behaviors affect healthcare professionals’ WTH, and which characteristics can promote the WTH of healthcare professionals.

A salient example occurred during the COVID-19 pandemic. Due to a reduced workforce and the increased number of hospitalized COVID-19 patients, questions were raised by various stakeholders (e.g., journalists, politicians, or physicians) regarding whether healthcare professionals should treat non-vaccinated and vaccinated patients equally (e.g., Hadrich, 2021; Khullar, 2021; Lacombe, 2021; Marcus, 2021; Rogers, 2021). These debates highlight the need to understand what factors shape healthcare professionals’ intentions to help, and what factors may counteract potential biases.

## Empathy: A Factor Influencing WTH?

The scientific literature generally divides empathy into two main domains, affective and cognitive empathy. The former refers to the ability to feel what others feel (Bryant, 1982), and the latter the capacity to comprehend others’ feelings (Hogan, 1969). Empathy, as a core skill in clinical settings (Nasello & Triffaux, 2023), is likely a significant factor that influences helping behaviors and the WTH among (future) healthcare professionals, as supported by the empathy-altruism hypothesis (Batson, 1987; Batson, 2011; Batson et al., 2014) and a recent meta-analysis (Yin & Wang, 2023). Baston’s hypothesis (Batson, 1987; Batson, 2011; Batson et al., 2014) states that empathic concern produces altruistic motivation that leads to prosocial behaviors (Batson, 2011, p.1).

In clinical settings, empathy in healthcare professionals is linked to increased patient satisfaction (Derksen et al., 2013; Hojat et al., 2011; Pollak et al., 2011), better treatment adherence (Attar & Chandramani, 2012; Zolnierek & DiMatteo, 2009), and improved clinical outcomes (Hojat et al., 2011). Thus, fostering empathy among healthcare providers is a major objective for improving quality of care.

However, it remains unclear how patients’ own behaviors — especially those perceived as unhealthy or responsibility-laden — affect professionals’ WTH, and whether empathy modulates this relationship.

## The Present Study’s Objectives

As demonstrated by the *Theory of Planned Behavior and Reasoned Action* (Ajzen, 1985; Ajzen, 1991; Fishbein & Ajzen, 1975), attitudes influence intentions that, in turn, impact behaviors. In the present study, we shift the paradigm by seeking to determine whether specific features from others predict WTH (which is compared to intentions). In the present study, we focused on prior behaviors adopted by patients (i.e., healthy or unhealthy behaviors) and on future healthcare professionals’ WTH. Therefore, we aim to answer the following question: are future healthcare professionals’ WTH significantly affected when patients exhibit healthy and unhealthy behaviors?

Furthermore, given the clinical importance of empathy and results from a meta-analysis showing that empathy is positively associated with WTH (Yin & Wang, 2023), we expected some positive associations between these factors. However, we sought to clarify which domain of empathy is specifically associated with WTH and whether healthy and unhealthy WTH scenarios affect the associations between empathy domains and WTH.

As peripheral objectives, we will determine whether grade (Bachelor versus Master students) impacts empathy and WTH and whether age similarly affects these two components. Finally, several studies showed that empathy levels significantly differed between men and women (Baron-Cohen & Wheelwright, 2004; Jolliffe & Farrington, 2006; Nasello & Triffaux, 2020; Triffaux et al., 2019; Triffaux et al., 2023). Thus, we expect to find a significant association between gender and empathy.

Regarding the association between gender, age, and WTH, we expect to find insignificant associations between these variables, as found by Hajdu et al. (2022).

However, the literature on gender differences in actual prosocial behavior remains mixed, with studies reporting both higher prosociality in women (Carlo et al., 2016; Perenc & Pezczkowski, 2018) and greater helping behavior in men (Eagly & Crowley, 1986).

## Method and Material

### Participants

A total of 301 respondents voluntarily completed the online survey, and one participant was excluded because s/he was not enrolled in a healthcare curriculum. Therefore, the final sample comprised 300 participants (82% women; *M_age_* = 23.5; *SD* = 5.97). Most were Bachelor’s students (*n* = 215), with Master’s students representing 85 participants, and mainly came from nursing (35%), physiotherapy (26%), psychology (9%), and medicine (8%) departments (see Table A in Nasello, 2025 for descriptive results). Most participants identified as Caucasians (*n* = 265) and reported an average parental socioeconomic status (64%). The financially independent students (*n* = 51, 39%) reported a low socioeconomic status (income < €1200/month).

Inclusion criteria required enrollment in a healthcare program, completion of at least one clinical internship, and age ≥ 18 years. Ethical approval was obtained from the Ethics Committee of the Department of Psychology at the University of Liège (Belgium), Reference N°: 2122-040. Informed consent was obtained online.

A power analysis conducted with G*Power 3.1.9.7 (Faul et al., 2007) determined that we would require at least 167 participants for our main analysis to achieve a power of 0.80 with an α-error settled at 0.05, 8 degrees of freedom, and to detect moderate effect sizes (settled at 0.3). The online data file is available at Nasello (2025).

### Instruments

#### Demographic Information

The student participants were asked to provide for their age, gender, ethnicity, year of study, department, the perceived economic status of their parents (when they were not financially independent), and their personal economic status when they were financially independent.

#### The Willingness to Help

Inspired by Koster (2007) and Koehler and Weber (2018), we designed 12 small scenarios describing clinical situations. There were six healthy and six unhealthy scenarios randomly distributed. The scenarios were structurally equivalent (see Tables B and C in Nasello, 2025). In the healthy scenarios, the protagonist (a patient) exhibited healthy behaviors but still got sick (e.g., A patient who does not smoke is diagnosed with lung cancer and is admitted to your department.). In contrast, in the unhealthy scenarios, the protagonist exhibited unhealthy behaviors and still got sick (e.g., A patient who smokes is diagnosed with lung cancer and continues smoking while being admitted to your department.). Each scenario included the following statement: “*In each of the situations, the target person is aware of their diagnosis or condition.*” Participants rated six items after each vignette using a 5-point Likert scale (1 = *Strongly disagree*; 5 = *Strongly agree*):

1. “I would provide optimal care to this person.”
2. “I would do my best to improve this person’s pathology.”
3. “I would visit this person several times (even more than recommended) to see if everything is going well.”
4. “I would come more quickly when this person calls for help.”
5. “I would persuade my colleagues that this person needs specific attention.”
6. “I would ask very specific questions to this person to better understand their suffering.”

Participants answered these questions using a 5-point Likert scale ranging from 1 (“Totally disagree”) to 5 (“Totally agree”). A global WTH score and two sub-scores (healthy vs. unhealthy scenarios) were computed. Internal consistency was excellent (α = .95 for both scenario types).

#### Empathy

The Basic Empathy Scale (BES, Jolliffe & Farrington, 2006; French version, D’Ambrosio et al., 2009) assesses two domains of empathy (affective and cognitive empathy) and four emotions (i.e., anger, fear, happiness, and sadness). We selected the BES over the Interpersonal Reactivity Index (Davis, 1983) and the Jefferson Scale of Physician Empathy (Hojat et al., 2002) because it uniquely focuses on emotional states rather than encompassing all mental states like its counterparts (see Jolliffe & Farrington, 2006 for more details). This allows for a more accurate targeting of the empathic processes involved in assessing their potential impact on WTH. The scale includes twenty self-report items and uses a 5-point Likert scale ranging from “Strongly disagree” to “Strongly agree.” The affective empathy domain consists of eleven questions, while the cognitive component has nine items. In this study, the internal consistency of the BES domains was found to be good (affective empathy: α = .83; cognitive empathy: α = .74).

### Data Analyses and Experimental Design

Structural equation modeling (SEM) was used to examine the relationships between empathy, WTH, and sociodemographic variables (gender, age, and grade). The model’s goodness of fit was assessed using five statistical indices: the chi-square goodness-of-fit (χ^2^), the Root Mean Square Error of Approximation (RMSEA), the Comparative Fit Index (CFI), the Tucker-Lewis Index (TLI), and the Standardized Root Mean Square Residual (SRMR). A non-significant chi-square value indicates a satisfactory fit of the model. However, since the chi-square test is influenced by sample size and can yield significant results with larger samples, a χ^2^*/df* ratio is used. The χ^2^*/df* ratio should not exceed 5 to display good goodness of fit. RMSEA, CFI, TLI, and SRMR were used to confirm the model’s fit (Schumacker & Lomax, 2010). CFI and TLI values greater than .9 indicate an adequate fit, while a value greater than .95 suggests a good fit. For the RMSEA and SRMR, a value below .08 indicates an adequate fit, while a value below .05 represents a good fit (Hu & Bentler, 1999).

A *t*-paired test and a correlation matrix accompanied the main analysis (SEM). The analyses were conducted using JAMOVI computer software, Version 1.6.23 (Jamovi Project, 2019) and R, Version 4.2.2 (R Core Team, 2021), *lavaan* package 0.6.15 (Rosseel, 2012).

## Results

### Descriptive Analyses and Correlation Matrix

Table 1 displays the descriptive statistics for all variables. A paired-samples t-test showed that WTH differed significantly across conditions, *t*_(299)_ = -5.62, *p* < .001, *d* = -.32: future healthcare professionals reported higher WTH toward patients who had engaged in healthy behaviors compared to those with unhealthy behaviors (small effect size).

**Table 1:** Descriptive Statistics

Variable	*M*	*Mdn*	*SD*	*Min*	*Max*
Age	23.5	22.0	5.97	18	53
AE	41.1	41.5	6.80	14	54
CE	37.4	38.0	4.21	23	45
WTH_UH_	137.9	139.0	18.07	76	180
WTH_H_	140.7	141.0	18.17	89	180
WTH_Tot._	278.6	280.5	35.23	179	360

*Note.* AE = Affective Empathy; CE = Cognitive Empathy; WTH_H_ = Willingness to Help, Healthy scenarios; WTH_UH_ = Willingness to Help, UnHealthy scenarios; WTH_Tot._ = Willingness to Help, all scenarios.

The correlation matrix (see Table 2) revealed that affective empathy was significantly correlated to WTH_H,_ *r* = .17; *p* < .01, WTH_UH,_ *r* = .18; *p* < .01, and WTH_Tot.,_ *r* = .18; *p* < .01. Cognitive empathy was also significantly correlated to WTH_H,_ *r* = .16; *p* < .01, but the correlation was stronger for WTH_UH_, *r* = .23; *p* < .001, and WTH_Tot.,_ *r* = .20; *p* < .01. Overall, the correlations between empathy and WTH domains were small.

**Table 2:** Correlation Matrix

	Gender	Age	Grade	AE	CE	WTH_UH_	WTH_H_	WTH_Tot._
Gender	—							
Age	-0.08	—						
Grade	0.065	-0.10	—					
AE	0.336***	-0.04	-0.07	—				
CE	0.099	0.054	-0.17**	0.385***	—			
WTH_UH_	0.141*	0.020	0.070	0.181**	0.225***	—		
WTH_H_	0.116*	0.012	0.119*	0.172**	0.161**	0.890***	—	
WTH_Tot._	0.132*	0.016	0.097	0.181**	0.198***	0.972***	0.972***	—

*Note.* AE = Affective Empathy; CE = Cognitive Empathy; WTH_H_ = Willingness to Help, Healthy scenarios; WTH_UH_ = Willingness to Help, UnHealthy scenarios; WTH_Tot._ = Willingness to Help, all scenarios.

*p < .05. **p < .01. ***p < .001.

Regarding the sociodemographic variables, gender (women were coded as 1) was significantly correlated to affective empathy, *r* = .34; *p* < .001, WTH_UH,_ *r* = .14; *p* < .05, WTH_H,_ *r* = .14; *p* < .05, and WTH_Tot.,_ *r* = .13; *p* < .05. Grade (Bachelors were coded as 1) was also significantly correlated to cognitive empathy, *r* = -.17; *p* < .01, and WTH_H,_ *r* = .12; *p* < .05. Finally, age was not significantly associated with empathy or WTH domains, *p*s > .05. All these correlations were small, except for the relationship between gender and affective empathy that is moderate.

### Structural Equation Modeling

**Figure 1:**
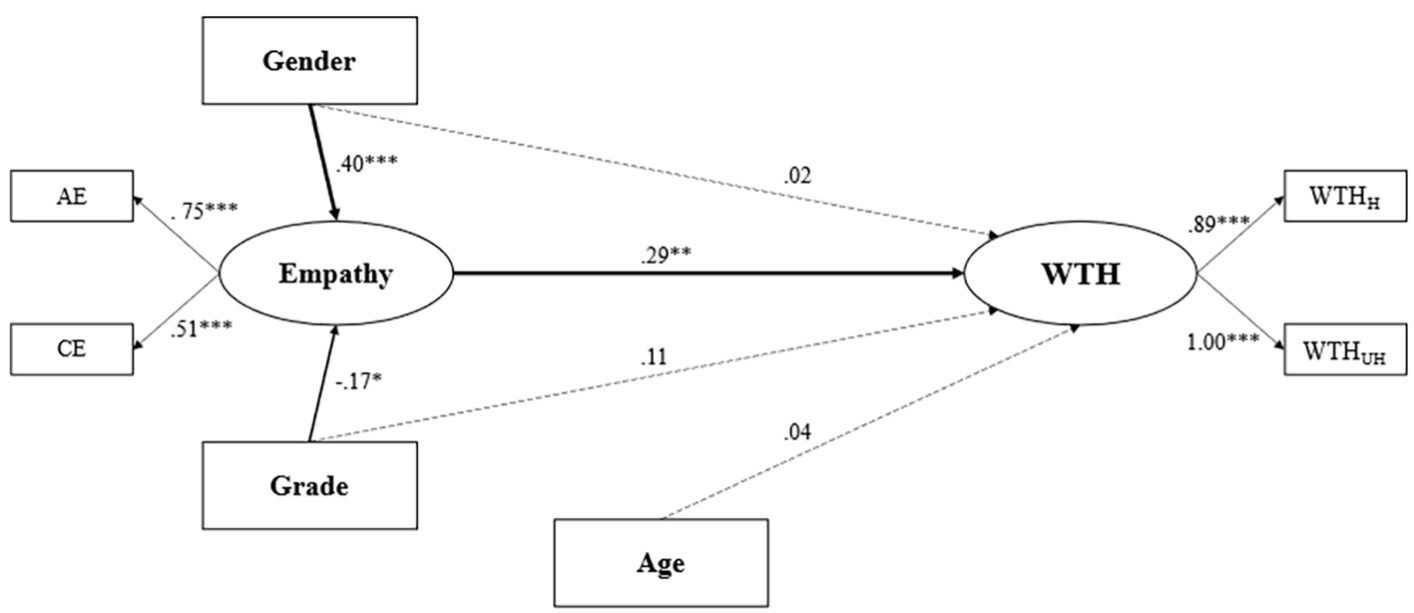
The Structural Model *Note.* Women and Bachelors were coded “1”. AE = Affective Empathy; CE = Cognitive Empathy; WTH_H_ = Willingness to Help, Healthy scenarios; WTH_UH_ = Willingness to Help, UnHealthy scenarios. *p < .05. **p < .01. ***p < .001.

The final model showed adequate goodness of fit indices, χ*^2^/df* = 2.71, *CFI* = .97, *TLI* = .95, *RMSEA* = .075, *SRMR* = .038. In this final model, we set the variance of WTH_UH_ at 1. As shown in Figure 1, empathy had a (moderate) significant direct link with WTH, β = .29; *p* < .005 (see Table D; Nasello, 2025), confirming the main hypothesis of this study. Gender, age, and grade did not significantly impact WTH, *p*s > .05.

Regarding empathy, we found that gender and grade had a significant direct link with empathy (gender: β = .40, *p* < .001; grade: β = -.17, *p* < .05; see Table D; Nasello, 2025). In other words, women presented higher levels of empathy than men (the effect size of this association is moderate) and Master’s students had higher empathy levels than Bachelor’s students (the effect size is small). Refer to Tables D–G in Nasello (2025) for complementary descriptive statistics in the Supplementary Materials.

## Discussion

Consistent with our expectations, the present study demonstrated a positive effect of empathy on WTH, which aligns with the findings of Yin and Wang (2023). Upon further analysis of this association, the correlation matrix revealed that future healthcare professionals with higher levels of affective and cognitive empathy were more inclined to assist patients exhibiting both healthy and unhealthy behaviors. In addition, when it came to patients with unhealthy behaviors, cognitive empathy was the strongest predictor of WTH. In other words, higher levels of cognitive empathy, and to a lesser extent affective empathy, are associated with a higher propensity to assist patients with damaging health behaviors.

However, a methodological point deserves emphasis. In this study, we utilized the Basic Empathy Scale (Jolliffe & Farrington, 2006), which focuses exclusively on the emotional dimension of empathy. Unlike the Interpersonal Reactivity Index (Davis, 1983), this scale does not assess broader mental states. Thus, the observed relationship between WTH and empathy indicates that it is primarily the ability to feel and understand others’ emotional states that matters. These findings are crucial, as we observed a significant decrease in WTH of future healthcare professionals when patients exhibited unhealthy behaviors compared to scenarios where patients displayed healthy behaviors.

Batson and colleagues (2014) explained that learning whether one’s help effectively alleviated the needs of a target is one of several circumstances that can impact altruistic behaviors. In light of our results, future healthcare professionals may anticipate a lower probability of achieving their desired goal (optimal patient recovery) when performing medical procedures. Consequently, they may exhibit less readiness to assist patients with unhealthy behaviors.

Furthermore, with regards to the impact of empathy on WTH, Batson’s empathy-altruism hypothesis (Batson, 1987, 2011; Batson et al., 2014) posits that individuals with higher levels of empathic concern experience an increase in altruistic motivation and are more inclined to provide targeted assistance to someone. However, it was unclear whether this prosocial behavior persisted regardless of the target’s personality traits or behaviors. 

The current study shed light on the fact that patients’ unhealthy or healthy behaviors do influence the willingness of future healthcare professionals to help but their empathy levels are also a significant (moderate) predictor. As mentioned, (1) both affective and cognitive empathy contribute to WTH, regardless of the patient’s behavior, and (2) cognitive empathy becomes especially influential when patients engage in unhealthy behaviors. These results highlight the protective value of cognitive empathy in maintaining professional engagement even when patients’ behaviors evoke negative judgments.

### The Role of Sociodemographic Variables

Regarding sociodemographic variables such as age, gender, or grade, none of these variables had a significant influence on WTH. This suggests that, across different levels of training, ages, and genders, future healthcare professionals express a similar baseline willingness to help. These results replicate the nonsignificant associations reported by Hajdu et al. (2022).

Regarding the effects of these sociodemographic variables on empathy, we found a significant and positive effect of gender: women exhibit higher levels of affective empathy. Additionally, we observed a significant negative effect of the grade variable: master’s students have higher levels of empathy.

The former result is a well-established empirical finding (Baron-Cohen & Wheelwright, 2004; Jolliffe & Farrington, 2006; Nasello et al., 2018; Nasello & Triffaux, 2020; Triffaux et al., 2019; Triffaux et al., 2023). Sex role stereotypes have been suggested as a potential explanation for the gender effect on empathy (Hoffman, 1977), but other authors argue that these gender differences are also present in elementary forms of empathy, such as mimicry, indicating a deeper-rooted effect (Goldman & Sripada, 2005; Hermans et al., 2006; Lakin et al., 2008; Schulte-Rüther et al., 2008).

The latter result may seem surprising because several studies have demonstrated a significant decline in empathy scores during education, particularly in the field of medicine (Hojat et al., 2004; Triffaux et al., 2019; Ward et al., 2012), as well as in various healthcare fields, including medicine (Nunes et al., 2011). However, the grade effect on empathy aligns with a recent study that revealed higher empathy scores among healthcare students (specifically, medical students) during the COVID-19 pandemic (Triffaux et al., 2023). The authors observed significantly higher scores in affective and cognitive empathy in the pandemic cohort compared to two pre-pandemic cohorts, using two different empathy measurement tools. The authors proposed that these higher scores observed in the pandemic cohort could be either transient or permanent. In either case, these higher scores could potentially have a detrimental effect (refer to Triffaux et al., 2023 for more details), sustaining the need to intervene at the medical curriculum level by proposing targeted courses on empathic skills.

### How to Implement the Present Findings in Education?

The study’s findings echo existing calls for structured training interventions into the education of future healthcare professionals to cultivate appropriate empathy skills (Hojat, 2016; Kilic et al., 2021; Nasello & Triffaux, 2020; Nasello & Triffaux, 2023; Riess & Kraft-Todd, 2014; Triffaux et al., 2019; Triffaux et al., 2023; Zhu et al., 2021). Such tailored interventions should prioritize the development of effective emotion regulation skills and the promotion of specific facets of empathy, such as empathic concern and perspective-taking (Nasello & Triffaux, 2023; Triffaux et al., 2023).

There are several ways to improve empathic skills. One model, proposed by Riess and Kraft-Todd (2014), is called E.M.P.A.T.H.Y. It focuses on nonverbal empathic skills and includes the following components:

**E**: Eye contact.
**M**: Muscles of facial expression.
**P**: Posture.
**A**: Affect.
**T**: Tone of voice.
**H**: Hearing the whole patient.
**Y**: Your response.

Another approach is Balint’s groups, where students participate as either a patient, a clinician, or an observer. This method helps develop facets such as empathic concern and perspective-taking (Zhu et al., 2021). Additionally, a new model has been proposed to understand the empathic process from the empathizer’s perspective. This model can shed light on how both maladaptive and adaptive responses occur, as well as how empathy can be associated with psychopathological symptoms (Nasello & Triffaux, 2023).

Alongside proposals aimed at developing relational skills centered on empathy, other approaches could be suggested and adapted for (future) healthcare professionals. For instance, authors such as Bianco and Castelli (2023), as well as Bateman and Fonagy (2019), have put forward various models intended to foster theory of mind and mentalizing capacities. Although these models have not yet been applied with the explicit goal of enhancing the interpersonal abilities of (future) healthcare professionals, they show considerable promise, particularly due to their clear proximity to cognitive empathy.

Bianco and Castelli (2023), for example, have identified three key components for cultivating theory of mind skills (i.e., the ability to attribute mental states to ourselves and others in order to make sense of people’s social behavior). These components include:

1. *Training second-order recursive thinking*, that is, the capacity to mentally revisit an event with the aim of recognizing that one might hold a false belief about what others think.
2. *Practicing the application of theory of mind principles*, which involves accurately identifying others’ mental states by formulating the most plausible interpretations in a given situation.
3. *Mentalizing thoughts and emotions*, referring to the ability to reason about both one’s own and others’ mental states while simultaneously taking into account both cognitive and affective aspects — understanding that these internal contents shape behaviors and subjective experiences.

These three components can be meaningfully linked to the framework proposed by Bateman and Fonagy (2019), who highlight how neural dynamics jointly enable the mind to:

1. Distinguish external reality from internal psychological experience and fantasy.
2. Support the development of an embodied sense of self, capable of acting as an intentional agent.
3. Perceive social interactions as meaningful.
4. Adopt others’ perspectives and, more broadly, engage in imaginative reasoning.

### Conclusion

The present study demonstrates that future healthcare professionals’ willingness to help varies depending on patients’ prior health behaviors, with lower WTH observed toward patients who engaged in unhealthy behaviors. Additionally, the findings provided strong evidence of empathy playing an increasing role, with a moderate effect size, in shaping the WTH among future healthcare professionals. Hence, our findings suggest that the *Theory of Planned Behavior and Reasoned Action* may require refinement. Indeed, attitudes and subjective norms will have a significant impact on the intention of future caregivers to help and care for a patient, as will perceived behavioral control and conscious decision-making. However, on the one hand, we see that certain individual characteristics of these future caregivers (their empathy) also play a significant role in their intentions to help, and on the other hand, patients’ prior health behaviors may influence how they receive care.

Overall, our results advocate for the promotion of empathic skills and related aspects within the academic curriculum of future healthcare professionals (e.g., incorporating targeted courses on empathic or mentalization skills). Addressing this challenge in education is crucial and ranks among the most fundamental tasks for the forthcoming decades.

The study’s main limitation lies in the fact that we measure WTH but not the actual helping behavior itself. Although it is likely that WTH translates into helping behaviors, this point remains hypothetical.

## Supplementary Materials

**Table:** 

Type of supplementary materials	Availability/Access
Data	
WTH and EMP data.	Nasello (2025)
Code	
Code R - Structure WTH.	Nasello (2025)
Material	
Basic Empathy Scale.	Nasello (2025)
Basic Empathy Scale - English.	Nasello (2025)
Information - Data and coding text document.	Nasello (2025)
Supp. Mats word document.	Nasello (2025)
Study/Analysis preregistration	
The study was not preregistered.	—
Other	
No other material to report.	—

## Data Availability

The online data file is available on the Open Science Foundation at Nasello (2025).
